# Hydrophobic pinning with copper nanowhiskers leads to bactericidal properties

**DOI:** 10.1371/journal.pone.0175428

**Published:** 2017-04-11

**Authors:** Ajay Vikram Singh, Semanur Baylan, Byung-Wook Park, Gunther Richter, Metin Sitti

**Affiliations:** 1 Physical Intelligence Department, Max Planck Institute for Intelligent Systems, Stuttgart, Germany; 2 CSF Thin Films Group, Max Planck Institute for Intelligent Systems, Stuttgart, Germany; University of British Columbia, CANADA

## Abstract

The considerable morbidity associated with hospitalized patients and clinics in developed countries due to biofilm formation on biomedical implants and surgical instruments is a heavy economic burden. An alternative to chemically treated surfaces for bactericidal activity started emerging from micro/nanoscale topographical cues in the last decade. Here, we demonstrate a putative antibacterial surface using copper nanowhiskers deposited by molecular beam epitaxy. Furthermore, the control of biological response is based on hydrophobic pinning of water droplets in the Wenzel regime, causing mechanical injury and cell death. Scanning electron microscopy images revealed the details of the surface morphology and non-contact mode laser scanning of the surface revealed the microtopography-associated quantitative parameters. Introducing the bacterial culture over nanowhiskers produces mechanical injury to cells, leading to a reduction in cell density over time due to local pinning of culture medium to whisker surfaces. Extended culture to 72 hours to observe biofilm formation revealed biofilm inhibition with scattered microcolonies and significantly reduced biovolume on nanowhiskers. Therefore, surfaces patterned with copper nanowhiskers can serve as potential antibiofilm surfaces. The topography-based antibacterial surfaces introduce a novel prospect in developing mechanoresponsive nanobiomaterials to reduce the risk of medical device biofilm-associated infections, contrary to chemical leaching of copper as a traditional bactericidal agent.

## Introduction

Microbial contamination of biomedical devices and implants due to biofilms remains a worldwide challenge and research interest. Progress in biomedical engineering has improved clinical outcome, on the other hand, the growing burden of biofilms has led to multidrug resistance (MDR) [1, 2]. Extracellular matrix (ECM) protected microbial colonies in biofilms need more than 1000 fold higher dose of antibiotics to be effective against the infections [3]. Implant replacement are often performed to remove biofilms and microbial payload to avoid patient mortality, thus adding economic burden to national health services. Recently, physical modification of medically relevant surfaces has emerged as a promising alternative to antibacterial chemical conditioning and antibacterial therapeutic agents among researchers and clinicians. Micro/nanoscale topography proves to be a low cost alternative for reducing bacterial colonization and biofilm formation on implants and their associated infections due to recent progress in micro- and nanoscale fabrication techniques [4].

Topographically patterned high aspect ratio nanomaterials are a unique class of biomaterials that are compatible with biomedical applications due to their superior biological response and strong hydrophobicity [5]. They help with tuning protein-cell-surface interactions to promote mammalian cell adhesion while reducing bacterial colonization [5, 6]. Contact killing due to mechanical injury and antibiofouling, i.e., inhibition of bacterial adhesion upon contact, are two mechanisms that may be exploited to reduce colonization on micro/nanopatterned surfaces. Bio-inspired Sharklet micropatterns are an attractive inspiration for designing such clinically-relevant antibacterial surfaces [7] to inhibit colonization of deadly *Pseudomonas aeruginosa* and methicillin-resistant *Staphylococcus aureus* (MRSA) biofilms [8]. Diverse materials, a variety of micro/nanopatterning techniques, and bio-inspired surface mimesis have been proposed to study the role of physical cues in bacterial colonization [9]. In particular, high-aspect-ratio nanoprotrusions with micrometer range spacing on different biomaterial surfaces were found to be highly bactericidal against both Gram-positive and Gram-negative bacteria [10]. Therefore, testing new materials and topography as antibacterial for biomedical applications is of great interest, since it may potentially reduce the biofilm on clinical devices.

Metallic copper (Cu) has long been utilized as a natural antibacterial coating on household utensils due to the coating’s ability to destroy a wide spectrum of microorganisms [11]. However, Cu-based surfaces with defined topography are rarely studied as antibacterial surfaces. Here, we report fabrication as well as an antibacterial study of Cu and Cu-modified gold (Au) as putative, clinically relevant surfaces to prevent bacterial colonization. We found hydrophobic pinning of water droplets in the Wenzel regime on nanowhiskers, enhancing *E*. *coli* killing via increased mechanical injury. Long-term cultures also demonstrate the ability to reduce bacterial biovolume on topographically modified surfaces compared with controlled control Cu surfaces.

## Materials and methods

### Preparation of copper and gold-shelled copper nanowhiskers

Si/SiO_2_/Si_3_N_4_ substrates were cleaned in the ultrasonic bath with acetone and isopropyl alcohol for 10 minutes, respectively. The substrate was coated with a 30 nm carbon (C) layer by magnetron sputtering physical vapor deposition (PVD) at room temperature (RT), since C is necessary to grow nanowhiskers. Afterwards, they were transferred into the molecular beam epitaxy (MBE) chamber, which is under ultra-high vacuum (UHV) condition (base pressure < 10^−9^ mbar) and Cu nanowhiskers were grown on the samples. The substrate temperature was 650°C and the rate of the Cu deposition was 0.05 nm/second. The holder, where the substrate is placed, was rotated during the deposition process. For Cu-Au core-shell nanowhiskers, the same procedure was applied to produce the Cu nanowhiskers but on a different substrate, partially coated with tungsten (W). Subsequently the Au shell was deposited onto Cu nanowhiskers with MBE at RT with the deposition rate of 0.01 nm/second (S1 Fig). Au and Cu were evaporated from effusion cells.

### Surface topography characterization

Nanometer-level profile, roughness, and film thickness data were characterized with Keyence VK-X Series 3D Laser Scanning Confocal Microscope, which automatically measures and analyzes 3D surfaces in non-contact mode.

### Transmission electron microscopy (TEM) analysis

For TEM imaging, Cu whiskers were scrapped over Mo TEM grid (Quantifoil, Holey carbon), and analyzed using Philips CM 200, operated at an accelerating voltage of 200 kV.

### Scanning electron microscopy (SEM) analysis

Cells were cultured on polydimethylsiloxane (PDMS) coupons and fixed using 2.5% of glutaldehyde with PBS for 6 hours. After fixation, coupons were rinsed two times in phosphate buffer saline (PBS) and then dehydrated in an ethanol series of 25%, 50%, 75%, 95%, and 100% (vol/vol) ethanol (dilutions were in deionized water) for 10 minutes each. Samples were then critical point-dried, mounted on aluminum stubs with carbon tape, and sputter coated with Au. Conductive paths were painted with colloidal silver. Micrographs were obtained on a Zeiss Supra 55VP FE-SEM using a secondary electron Everhart–Thornley or in-lens detector.

### Water contact angle and zeta potential measurement

Contact angle measurement was performed using a Kruss Goniometer (Kruss GmbH, Germany) with the ADVANCE™ spherical drop analyzer. Ultrapure water droplets (Millipore) were generated with 2 μL dose with 0.2 mL/minute and multiple measurements for 5 seconds were carried out at 1 frames per second (fps) camera recording. The tangent fit method was adopted for baseline adjustment during tilting, advancing and receding contact angle measurement. Zeta potential was determined via streaming current and streaming potential measurement using SurPASS electrokinetic analyzer (Anton Paar Company). A rectangular nanowhisker sample (1 cm x 1 cm x 0.2 cm) was inserted into adjustable gap cell with a holder in the 75–140 μm spacing range under a pressure of 0.3 bar. Samples were rinsed for 150 seconds under maximum pressure of 0.3 bar and subsequently streaming current was measured for 20 seconds under the same pressure. The Helmholtz–Smoluchowski equation was used establish zeta potential as a function of the pH value under auto-titration capability [12].

### Bacterial strains and culture conditions

Gram-negative *Escherichia coli* (*E*. *coli)* MG1655 (ATCC700926) was inoculated from glycerol stock into motility medium (0.01 M potassium phosphate, 0.067 M sodium chloride, 10^−4^ M EDTA, 0.01 M glucose, and 0.002% Tween-20, pH 7.0) and grown overnight at 37°C in a shaker incubator. The bacteria were subsequently stained with SYTO9 (ex/em 485/498) by mixing the dye (prepared as per instructions from the vendor) and the bacterial suspension 1:1, incubating for 15 minutes and gently washing the cells three times with PBS to remove excess SYTO9. Prior to seeding, samples were sterilized via UV treatment into a biological safety cabinet. Sterile whisker and control in culture plates were inoculated with 20 μL fresh culture diluted at a ratio of 1∶90 in Dulbecco's Modified Eagles Medium without antibiotic (DMEM, Hyclone; Gibco). Simulating the biofilm formation in a real biological environment, samples were left at 30°C for 48 hours with constant shaking at 200 rpm to prevent settling of the cell solution in five independent experiments in triplicate. For bacterial adhesion, cells were collected at the logarithmic stage of growth and seeded on the whisker and control surfaces. After every 2 hours samples were stained with live/dead cell assay kit (Baclight, Invitrogen) and visualized using confocal laser scanning microscopy (CLSM). The samples for microscopic imaging were prepared by standard procedures with optimum care to avoid any modification to the distribution and the orientation of bacteria over the surface, influencing cell parameter quantification or cell retention on the surface. This is important because bacteria imaging results can be affected by the hydrodynamic conditions. Therefore, the methods of fixation and drying of the cells for CLSM and atomic force microscope (AFM) imaging have been confirmed prior to sampling.

### Quantification of bacterial density and biofilm formation on a flat nanowhisker surface

Bacterial density (total bacteria colonies) over the nanostructured surfaces was determined by summing up the number of live and dead bacteria colonies quantified using ImageJ. In order to image the viable bacterial count and the extracellular polymeric substance (EPS), established microbial staining techniques were adopted. For live and dead bacterial count, after 24 hours incubation of bacteria on whisker and control sample, the substrates were rinsed twice with Tris-buffered saline (TBS) comprised of 42 mM Tris–HCl, 8 mM Tris Base, and 0.15 M NaCl (Sigma Aldrich). Then incubated for 15 minutes in dark with the BacLight Live/Dead solution (Molecular Probes Inc., Germany) dissolved in TBS at the concentration recommended by the manufacturer, 50% glycerol solution in TBS, visualized and counted *in-situ* using Confocal Laser Scanning Microscopy microscope (Nikon Eclipse Ti confocal microscope with Yokogawa CSU-W1 spinning disk) with a water immersion objective lens at 40x magnification, zoom 1∶5 and image analysis were performed with ImageJ NIH image processing software. For the biofilm, after 24 hours of adhesion at RT, the substrate was rinsed with 150 mM NaCl in order to eliminate any non-adherent bacteria before and 250 μL TBS was further added to the culture. All of the different substrates were incubated for 24 hours at RT. After the development of biofilms, the substrates were rinsed with 150 mM NaCl and 5 μM of Syto9 were added to TBS containing a cell permeable green fluorescent nucleic acid marker. The culture plate with the sample was then incubated in the dark at RT for 30 minutes to enable the fluorescent labeling of the attached bacterial cells. Samples were visualized and biofilm structural properties were quantified in situ using CLSM at 40x magnification of water immersion objective lens with a 0.8 N.A., zoom 1∶5. The average z-stacks of one μm were acquired from each biofilm horizontal plane with a maximum of five stacks at a different field of view with a piezo scanner. 3D projections of biofilms structure were reconstructed using the Nikon NIS core software. The quantification of biovolume of encapsulated bacterial cells in EPS matrix, representing the overall volume of cells in biofilm (μm^3^), was carried out using free PHLIP software.

### Statistical analysis

Values are reported in the text as value ± SD. For statistical comparisons between groups with normal distributions, Student’s two-tailed t test was used. Error bars in Fig are either SD or standard error of the mean, as indicated in the legends. P-value of <0.05 was considered to be statistically significant. For the statistical validation of data, three independent experiments (n = 3) were performed in triplicate.

## Results

### Fabrication of nanowhiskers and surface topography characterization

**Fig 1:**
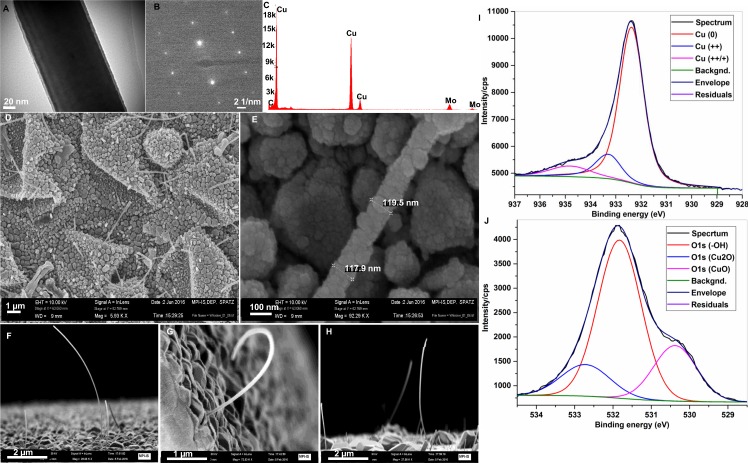
Microscopic examinations of Cu and Au-coated Cu whiskers. (A-C) Transmission electron microscopy micrograph (A), diffraction pattern (B) and energy dispersive x-ray analysis showing material properties of Cu whiskers (C). (D-E) Low and high magnification view of Cu nanowhiskers. (F-H) SEM micrograph at a tilted angle showing high aspect ratio Cu whiskers. (I-J). Deconvoluted XPS spectra showing the elemental composition of Cu and oxygen of nanowhiskers.

Single-crystalline, high-aspect-ratio Cu nanowhiskers were grown under UHV conditions by MBE on C-coated Si/SiO_2_/Si_3_N_4_ substrates at 650°C. Carbon coating and elevated surface temperature are essential for the growth of nanowhiskers [13]. The Au shell was deposited onto Cu nanowhiskers with MBE at RT with a deposition rate of 0.01 nm/second. A representative bright field transmission electron microscopy image of a 160–170 nm diameter Cu whisker with selected area electron diffraction (SAED) pattern is shown in Fig 1A and 1B, respectively. The diffraction pattern can be indexed to fcc Cu in the [112] zone axis. The thin halo or shell visible in the bright field TEM micrograph is the oxide layer formed on the Cu nanowhiskers after exposure to ambient atmosphere. Energy dispersive analysis of x-ray (EDAX) clearly shows materials property of the substrate and Mo signals comes from the TEM grid (Fig 1C).

Fig 1D and 1E are magnified top-view SEM images. The morphology and topography of tilted-angle nanowhiskers are shown in panels F-H, these protrude from the surface and are distributed heterogeneously. S1A and S2B Figs demonstrate the length and diameter distribution of nanowhiskers, respectively. Using x-ray photoelectron spectroscopy (XPS), we quantitatively studied the elemental composition of the Cu surfaces to understand the oxidation state and Cu-O stoichiometry. As shown in Table 1 and Fig 1I and 1J, peak binding energy calculations predominately indicate major fraction of metallic Cu and minor fraction of CuO/Cu_2_O oxidation states [14, 15]. O1S peak BE~531 indicates Cu surface associated -OH functional group. XPS analysis of Au-shelled Cu whisker show metallic Au as major composition with a small fraction with Au +/++ gold oxidation state with a binding energy of 84 and 87 eV associated with Au 5f_7/2_ and 4f_5/2_ photoelectron lines.

**Table 1:** XPS surface composition and elemental analysis of Cu and Au-shelled Cu whiskers.

	Cu 2p_3/2_	O_1_s
*Metallic Cu*	932.38 eV	
*CuO*	933.29 eV	530.38 eV
*Cu*_*2*_*O*	934.8 eV	532.72 eV
	**Au4f7/2**	**Au 4f5/2**
*Metallic Au*	83.80	87.85
*Au +/++*	84.16	87.81

**Fig 2:**
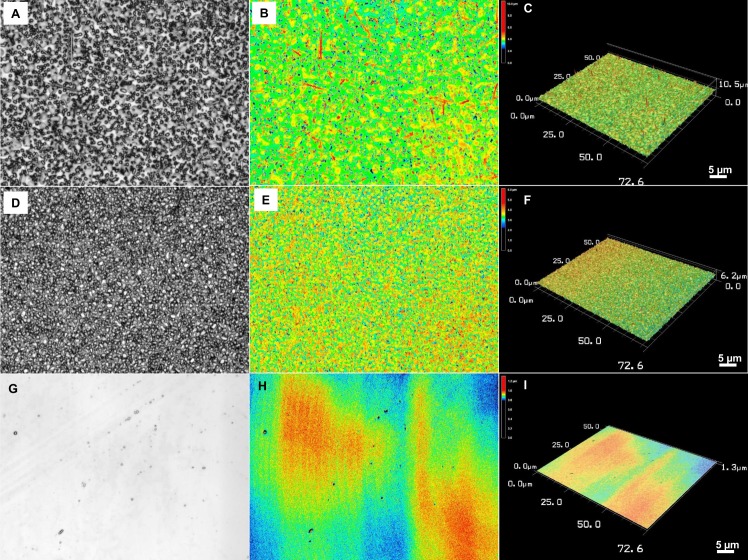
Qualitative and quantitative characterization of surface topography with laser scanning microscopy. Optical, height and 3D topography of Au-coated Cu nanowhiskers (A-C), Cu whiskers (D-F) and flat Cu surfaces (G-I).

We performed non-contact mode laser scanning of the surface to quantitatively assess the arithmetical mean height (Ra), max height (Rz), and texture aspect ratio (Str). These quantitative parameters determine the micro-nanoscale roughness of a material surface and dictate its wettability, which indirectly controls the biological interaction with material surfaces [16]. Fig 2 shows a laser-scanned optical image, the height, and three-dimensional (3D) topography of the two chemically different Cu surfaces along with control flat surface. A minor difference between Au-Cu and pure Cu nanoscale rough surfaces was qualitatively observed, however, substantial differences with the control surface were also evident (compare Fig 2A–2F with Fig 2E–2G; Table 2). The characterization of 3D topography over a 2D profile is important, since the latter may be limited in terms of the real surface topography. The former is useful with reference to cell-surface interactions in order to develop function systems for biological applications [17, 18]. Table 2 shows the detailed quantitative parameters with significance differences between arithmetic mean peak curvature of roughness parameters, which might be attributed to variations in texture shape versus direction connected versus isolated features in the *z*-direction) [19]. It is qualitatively visible as sparse on Au versus dense topography in laser scanned images shown in Fig 2A and 2D.

**Table 2:** Statistical analysis of 3D surface topography quantitative characteristics of the Cu films with microprotrusions and surface roughness.

	*Ra (μm)*	*Rz (μm)*	*Str (μm)*	*Spc (μm)*
*Au-shelled Cu whisker*	18.0 ± 0.2	6.50 ± 0.4	0.63 ± 0.02	26045.11 ± 101
*Cu whisker*	20.0 ± 0.1	4.19 ± 0.1	0.92 ± 0.05	69464.57 ± 176
*Flat thin Cu film*	0.03 ± 0.01	0.86 ± 0.06	0.06 ± 0.01	10481.52 ± 59

### Zeta potential and water contact angle measurement

**Fig 3:**
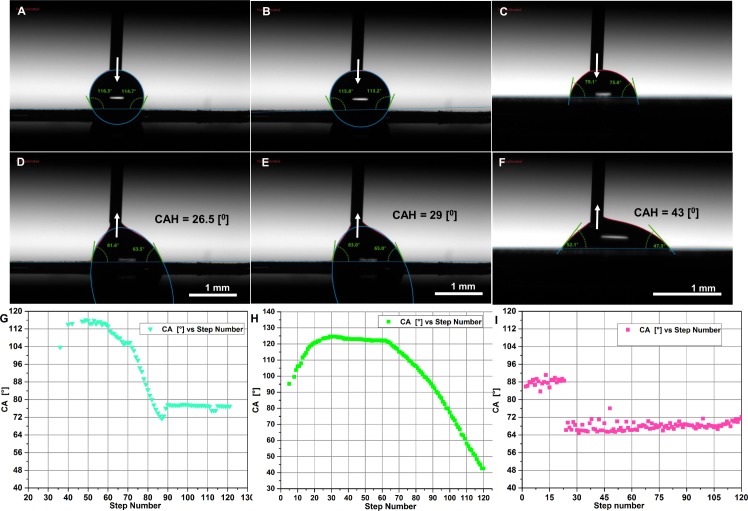
Comparison of wettability analysis of Cu whiskers versus control flat Cu thin films. Dynamic contact angle hysteresis (CAH) analysis via advancing (A-C) versus receding (D-F) contact angle of whisker and flat samples. (G-I) Mean contact versus step number surface profiles of whisker against the control flat surfaces.

We measured the zeta potential of the surface to understand the surface charge and electrokinetic response of the nanowhisker surface in context with wetting properties (S3A Fig). Water contact angle was determined and as shown in Table 2, static water contact angle on native Cu and Au-coated Cu nanowhisker films showed hydrophobic surface chemistry. However, flat Cu surfaces are hydrophilic in nature. Wenzel regime pinning of water droplets on nanowhisker surface was tested. This was achieved by dynamic tilting of the goniometer stage to 90°, adjusting tilt angle to 60°/minute and returning back to the initial stage at 120°/minute. The accompanying movie (S1 Movie) shows the pinning effect as a semi-spherical drop. The shape is deformed under gravity to a fan shape rim after tilting the stage. Due to localized pinning, the drop does not leave the surface. To test whether the pinning effect is also dominant over bigger droplets, the drop volume was increased from 2 μL to 10 μL. It was postulated the bigger droplet may fall apart from the pinned whisker surface due to increased weight, however the bigger droplets did not fall apart from the pinned surface as shown in S2 Movie. Conversely a flat Cu surface is hydrophobic (but less wettable). The 10-μL volume drop fell apart when dynamic tilting was applied due to the lack nanoscale protrusions, shown in S3 Movie. Fig 3A–3F show dynamic (advancing versus receding) contact angle hysteresis (CAH) of the droplet on the three fabricated films. A spherical shape of water droplets characterizes the advancing stage, whereas the receding stage is the opposite of the advancing stage with a slightly curved baseline. These two stages are quantitative indicators of hydrophobicity of the nanowhiskers [20]. The ellipse fitting of a water droplet advancing and receding state profiles of whisker films demonstrated a dynamic CAH of 26.5° and 29°, respectively. However, on flat surfaces the CAH was higher (about 43°) thereby confirming the inherent hydrophilicity of the whiskers substrate during dynamic hysteresis (Table 2).

### Bacterial interaction studies with whiskers

**Fig 4:**
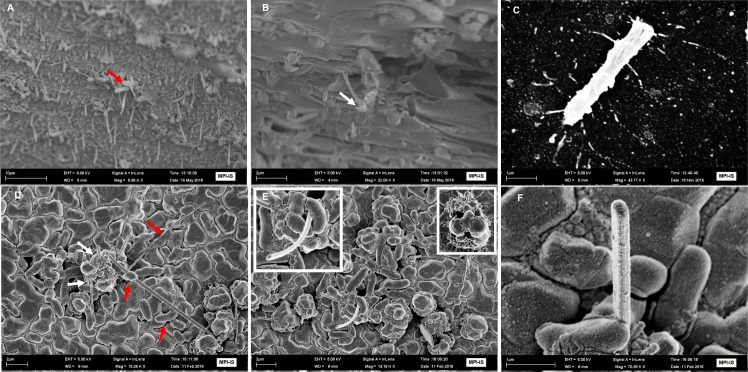
SEM micrograph showing bacterial interactions with whiskers and mechanical injury. (A-C) Low and high magnification SEM images showing whiskers piercing through the bacterial membrane on Cu whiskers (red versus white arrow exhibit low and high magnification scanned images at tilted angle. (D-F) Bacterial cluster “pinning” on high-aspect-ratio Au-coated Cu whiskers (white arrows in 4A). Red show a high aspect ratio piercing through many bacterial cell membrane at low (4B) and high magnification (4C). Insert in Fig 4B illustrates microcolonies pinned at whisker bottom.

The preference of bacterial adhesion to whiskers versus a flat Cu surface was investigated. The influence of a micrometer-scale surface comprising of sub-micrometer topography on bacterial adhesion was assessed. For this, a surface comprising of nanoscale whiskers topography was coated with *E*. *coli* MG1655 bacteria. The viability of bacteria was assessed by utilizing live-dead assays for 48 hours. Fig 4A–4F are SEM images showing Gram-negative *E*. *coli* bacteria attaching to the submicron-scale whiskers and pervade the spaces between vertical protrusions pinning bacterial culture media. This forcibly pushes bacterial cells to the surface. Additionally the whiskers reduce the local substrate surface area available to the bacterial cell body, however increase the overall surface area. It is also evident that *E*. *coli* preferentially enters spaces between two whiskers where the diameter is less that the bacterial cell body. This is in agreement with a previously published report [21].

**Fig 5:**
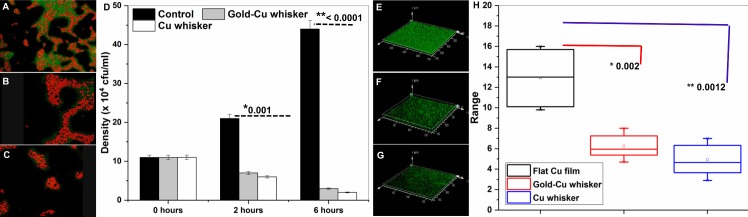
3D topographic reconstruction of CLSM image showing bacterial microcolonies encapsulated in EPS forming a thick biofilm on whisker with scattered patches of microcolonies on (stained green with B-35000, Backlight green live bacterial stain). Right panel depicts box and whisker diagram of bacterial biovolume of three samples. A box represents 25^th^ to 75^th^ percentile range, intersected by median line. Whiskers extend above and below the box range, indicating highest to lowest values.

Bacterial cells in contact with the whisker surface exhibit an altered phenotype compared to cells in contact with the Cu surface (see the SEM images in Fig 4A–4F; for comparison of surfaces, see Fig 4C, S3B and S4 Figs). Bacterial cells in direct contact with nanowhiskers are physically deformed, undergo rupturing and are non-viable due to the mechanical injury (Fig 4C). Energy dispersive x-ray and elemental analysis show nanowhiskers piercing and penetrating the *E*. *coli* membrane (S2C and S2E Fig). The live and dead populations of cells in contact with nanowhiskers was confirmed using CLSM. The extent of bacterial cell injury in the presence of nanowhiskers can be visualized in propidium iodide (PI) stained cells (fluorescent red) in Fig 5A. The PI staining of bacterial cells grown on a flat Cu surface was minimal (Fig 5B and 5C). Interestingly Syto9 and PI staining indicated that there was an equal density of live to dead cells covering a large surface area with respect to the control (whose surface was flat) (Fig 5A). In contrast to bacterial inhibition, mouse myoblast C2C12 cells interacting with nanowhiskers exhibited a stable trend as determined by total viable cell density quantification with MTT assay over 7 days (S3C Fig).

Total densities of test and control Cu films were quantified after 2 and 6 hours time points (Fig 5D and Table 3). Cu coating is known for its antibacterial properties. Mechanical injury was observed in nanowhiskers coated with Cu and Au-coated Cu nanowhiskers, irrespective of the biocompatible nature of Au. The reduction in cell viability is attributed to physical injury of Gram-negative *E*. *coli* [22–24]. Greater deformational stress is required to disrupt the thick bacterial cell walls from the outer-to-inner preface. In spite of this, Gram-negative *E*. *coli* cells show significant cell injury. The results clearly indicate the capacity of CuO whiskers to counteract bacterial survival strategies. The inactivation of bacteria due to mechanical injury due to Cu whiskers coated with and without Au nanowhiskers can be corroborated with previously published research [9]. The results clearly indicate that the deforming stress applied by Cu whiskers is substantial and may be used in counteracting bacterial survival strategies. Bacterial inactivation due to mechanical injury is comparable on Cu whiskers coated with and without Au nanowhiskers and is consistent with similar results published previously [25].

**Table 3:** Physicochemical and bacterial adhesion response on different nanowhisker samples.

*Topography*		*Au-coated Cu nanowhiskers*	*Cu Nanowhiskers*	*Flat Cu film*
*Physicochemical*	Static Wettability [°]	119 ± 12	116 ± 19	65 ± 08
	Dynamic Wettability (CAH) [°]	26.5 ± 06	29 ± 03	43 ± 10
	Chemistry	*Au_x_O_x_	*CuO_x_	CuO_x_
	Dimensions (mean ± sd)	12.2 ± 1 μm height with 0.135 ± 0.011 μm diameter	7.5 ± 0.6 μm height with 0.120 ± 0.029 μm diameter	0.05 μm homogenous thin film
*Bacteriological effectiveness*		Adhesion friendly, poor mechanical injury to *E*. *coli*	Adhesion friendly, high mechanical injury to *E*. *coli*	Adhesion friendly, Less mechanical injury *E*. *coli*
*Bacterial inactivation efficiency*	Cells inactivated in 2 hours incubations	3 x 10^3^ per cm^-2^ minute^-1^	3 x 10^4^ per cm^-2^ minute^-1^	significantly less cell inactivation over larger duration observed

* x = stoichiometry between 0.5–1

Fig 5D shows a column plot where bacterial density significantly increases after 2 hours while on a flat Cu film and reduced on a whiskers surface. The long-term adhesion potential and biofilm formation of different samples was further investigated. A thick carpet like biofilm of extracellular polymeric substance (immunostained light green) can be seen in the 3D reconstructed image in Fig 5E–5G. Quantitative analysis and characterization of structural parameters of the biofilms in different samples using CLSM are indicated as a biovolume (box and whiskers plot, Fig 5H). The stacked confocal images were analyzed as previously described. Whiskers with few, small-scattered cell clusters and big voids without colonies can be seen and agreement with live/dead assays (Fig 5A–5C). The reconstructed 3D biovolume in Fig 5E–5G exhibited similar trends and adopted a different morphology on the flat surface control.

The thickness of the biofilm on the control flat surface is due to the high amount of EPS produced by within the 3D matrix that harbors microcolonies of *E*. *coli*. The results obtained in the present study are in agreement with previously published work investigating EPS production by Gram-positive species on comparatively smoother titanium surfaces [26].

## Discussion

Sub-micron scale bumps and nanoscale geometry contribute to the difference in dynamic hysteresis between nanowhiskers and flat control surfaces. These hierarchical structures influence the hydrodynamic interaction of water drops and dissolved biomolecular entities, thereby affecting the capacity of surface bacterial colonization and biofilm formation [4, 27–31]. Additionally, the pinning behavior of water droplets on whisker topography versus flat control surfaces show hydrophobic droplets remain in the Wenzel regime [32].

Gram-negative cells are more prone to mechanical injury unlike Gram-positive cells, as they do not possess thick peptidoglycan cell wall. This cell wall makes the cells five-fold thicker, rigid, and resistant to mechanical lysis [33]. Such natural bactericidal surfaces are effective as antibacterial agents against Gram-negative bacteria. Their physical topography effectively overcomes the cell rigidity via penetration and lysis. This is in contrast to chemically- designed anti-biofouling surfaces which are effective against Gram-positive and Gram-negative bacteria [34].

There could be two regimes of nanowhisker-bacterial interactions on hydrophobic surfaces as discussed herein:

1. Hydrophobic surfaces with micro/nanostructures at the contact line (the Cassie-Baxter heterogeneous wetting regime). A liquid droplet with bacterial culture is placed on the substrate, which creates small air pockets underneath. The dynamic nature between the contact line and bacterial swimming, and tumbling at nanowhisker interface traps cells and disrupts the cell membrane.
2. Hydrophobic sharp micro/nanoscale rough surfaces with a stable equilibrium state (i.e., minimum free energy state for the system), where bacteria in liquid drops constantly interact with the contact line, receiving mechanical injury (the Wenzel heterogeneous wetting regime).

In order to confirm these two regimes, 10-μL drops were placed and forcefully shook. The *in-situ*–OH stretching signature of water molecules before and after shaking was assessed using infrared microscopy. The characteristic signature of–OH vibration from water was at 3450 nm approximately. The range of the broad peak is 3500–4000 nm and arises from water vapors due to the water drop being shaken off from the surface (S5 Fig). This indicates trapped water molecules in between whiskers with respect to the Wenzel regime [35]. The “pinning effect” influences the short-term viability of *E*. *coli*, where upon adhesion for couple of hours the bacterial cell become inactive (Table 2). Changing the surface chemistry of Cu whiskers by fabricating Au-shelled Cu whiskers does not alter the wettability and pinning effects. Bacterial cells are however susceptible to penetration and rupturing. In the long term, bacterial cells interacting with nanowhiskers are unable to form biofilms and have significantly lower biovolume compared to the flat control. The formation of biofilms is an important criterion for the successful invasion of bacterial cells on an implant surface [30]. The current findings demonstrate the possibility of tailoring surface morphology of biomedical prosthetics/implants to promote mammalian cell interactions while inhibiting bacterial colonization.

### Conclusions

MBE-fabricated Cu nanowhisker surfaces with micro/nanoscale roughness exhibit hydrophobicity with dynamic contact angle hysteresis in the Wenzel regime. This results in a “pinning effect of water drops” on nanowhiskers, which encouraged bacterial killing due to mechanical injury to the bacterial membrane. It is noteworthy to extend these experiments and investigate mammalian cell interactions with implants and prosthetics. Furthermore, it is important to explore molecular strategies for reducing broad range bacterial cell interaction and biofilm formation. Chemical leaching of Cu as a traditional bactericidal agent is beneficial; however, this current topography-based antibacterial study should pave the way for developing novel mechanoresponsive nanobiomaterials to reduce infection in a clinical setting.

## Supporting information

S1 Fig Schematic showing surface processing and fabrication strategy of Cu Nanowhiskers. (PDF)

S2 Fig Nanowhisker size distribution and bacterial EDAX analysis. (A-B) Nanowhisker length distribution and mean diameter quantification. (C-E) High magnification SEM micrograph showing bacterial cells over nanowhiskers, EDAX and elemental map.
(PDF)

S3 Fig Biochemical and toxicity analysis of nanowhiskers. A. Zeta potential measurement of nanowhiskers. B. Graph showing antimicrobial efficacy of nanowhiskers in terms of log reduction of CFU/mL. C. Assessing cytotoxicity of nanowhiskers to mouse myoblast C2C12 cell lines.
(PDF)

S4 Fig *E*.*coli* over control flat copper surface (insert shows low magnification view of SEM). (PDF)

S5 Fig Infrared spectroscopic analysis of residual water after pinning water drops at Cu Nanowhiskers. Blue line plot shows FTIR analysis before applying water drops, indicating atmospheric water vapors. Red curve represents FTIR spectra taken immediately after “pinned” water drop was forcibly shaken off the surface and spectra recorded. Black curve are recorded after 8 hours of samples were kept at room temperature. Reduced intensity exhibit some amount of residual water might had evaporated.
(PDF)

S1 Movie Deformation of water drops over Gold coated Cu whisker when stage was dynamically titled from 0^0^−90^0^. (AVI)

S2 Movie Deformation of water drops over Cu whisker when stage was dynamically titled from 0^0^−90^0^. (AVI)

S3 Movie Water drop buckling and falling apart over unpatterned flat surface when stage was dynamically titled from 0^0^−90^0^. (AVI)
