# Supplementary figures and images for: Hydrophobic pinning with copper nanowhiskers leads to bactericidal properties
Source: PLoS One. 2017 Apr 11;12(4):e0175428. doi: 10.1371/journal.pone.0175428 (PMC5388474; doi:10.1371/journal.pone.0175428)

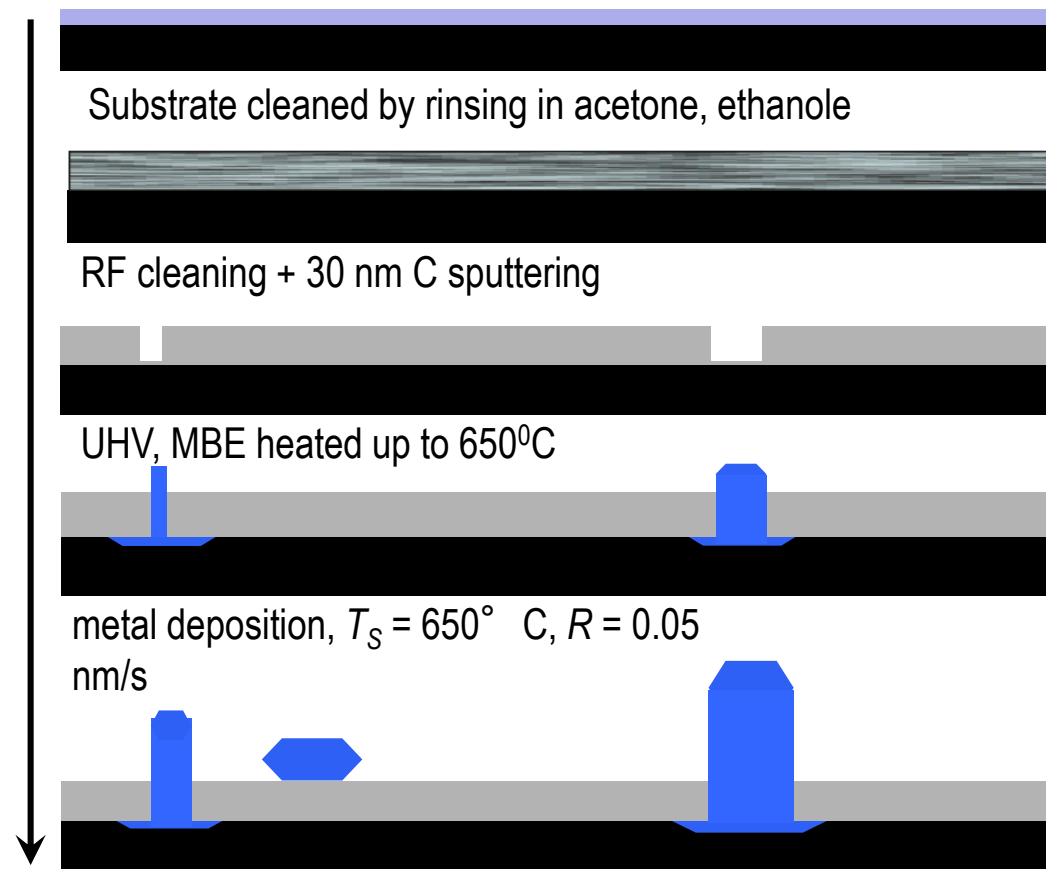

Supplement: S1 Fig — (PDF) [file pone.0175428.s001.pdf]

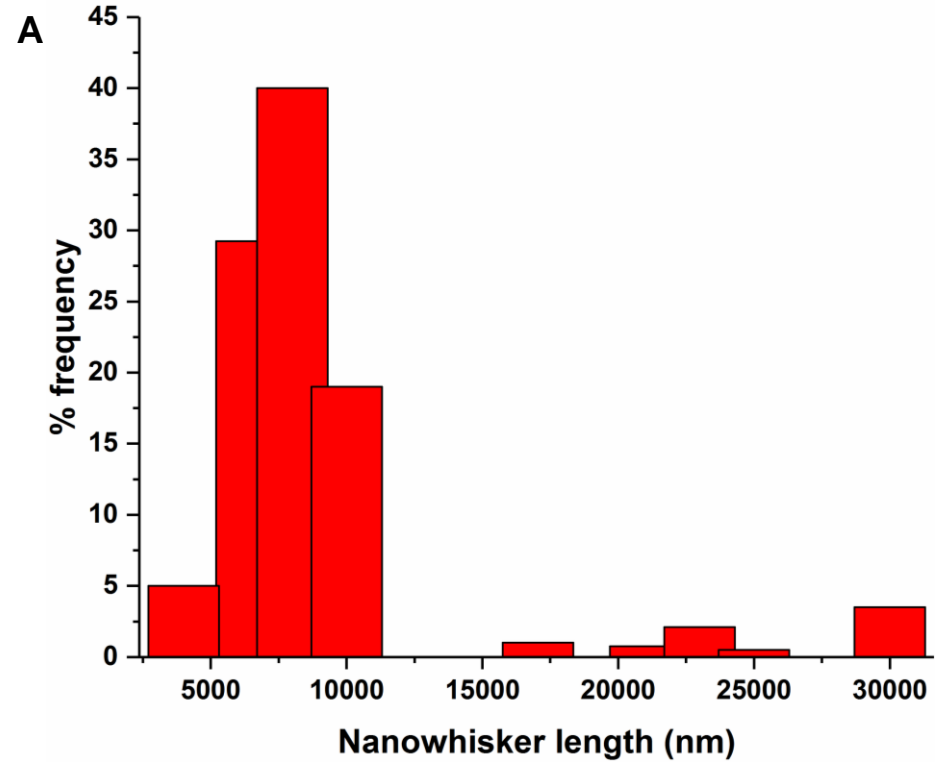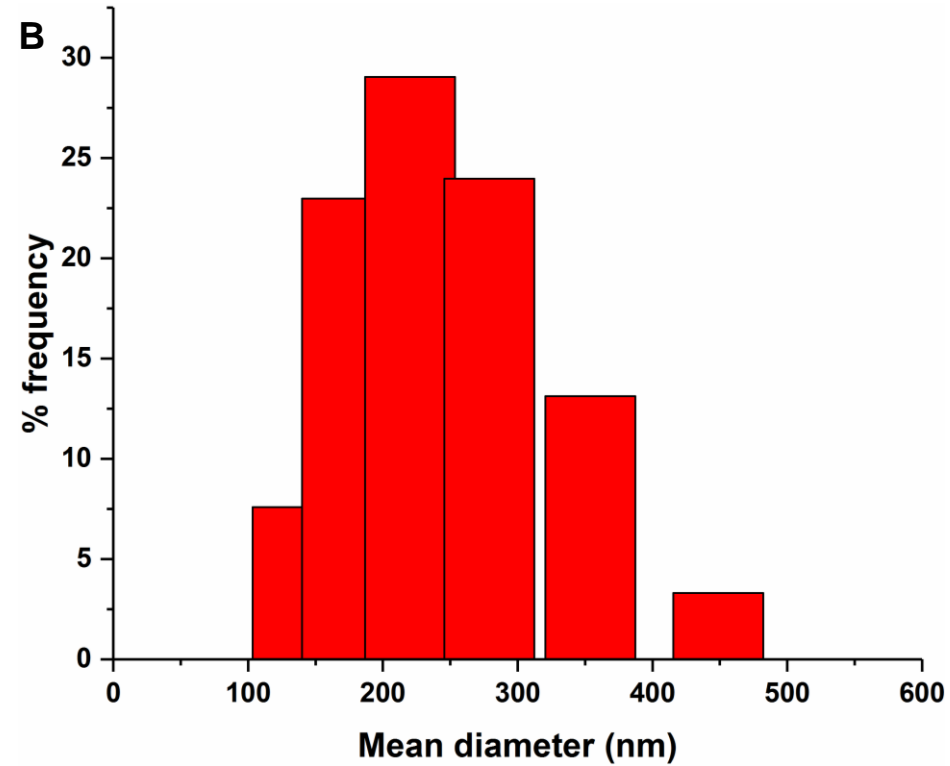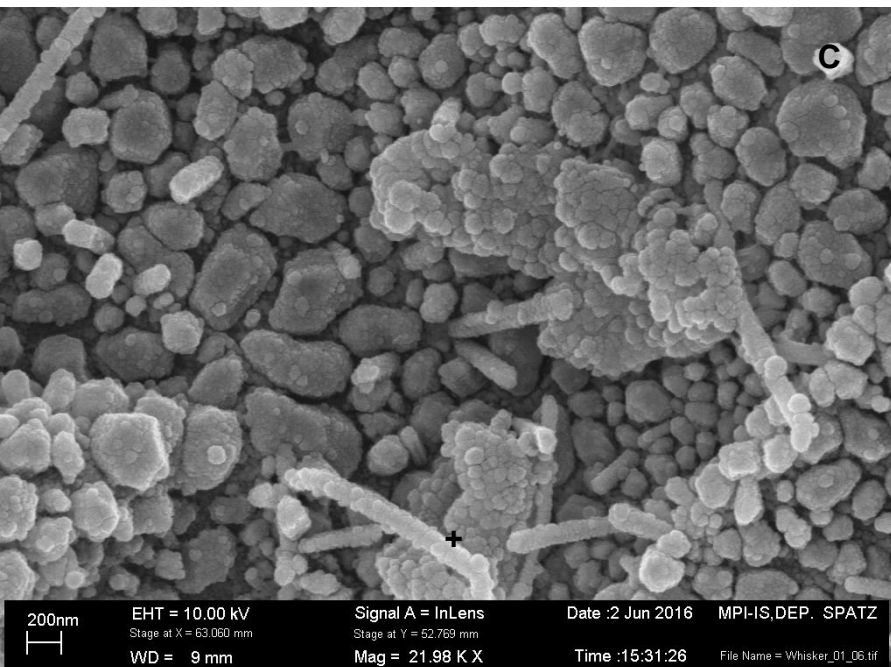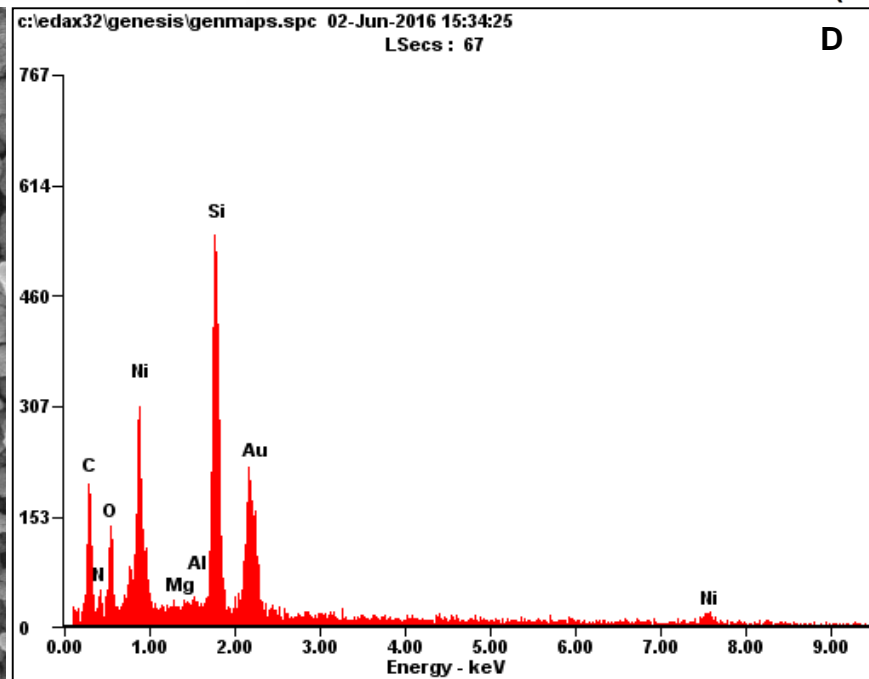

| <i>Element</i> | <i>Wt%</i> | <i>At%</i> | <b>E</b> |
|----------------|------------|------------|----------|
| <i>CK</i>      | 17.12      | 45.29      |          |
| <i>NK</i>      | 02.21      | 05.02      |          |
| <i>OK</i>      | 03.73      | 07.40      |          |
| <i>MgK</i>     | 00.13      | 00.18      |          |
| <i>AlK</i>     | 00.15      | 00.18      |          |
| <i>SiK</i>     | 15.88      | 17.96      |          |
| <i>AuM</i>     | 23.48      | 03.79      |          |
| <i>NiK</i>     | 37.30      | 20.19      |          |
| <i>Matrix</i>  | Correction | ZAF        |          |

Supplement: S2 Fig — (A-B) Nanowhisker length distribution and mean diameter quantification. (C-E) High magnification SEM micrograph showing bacterial cells over nanowhiskers, EDAX and elemental map. (PDF) [file pone.0175428.s002.pdf]

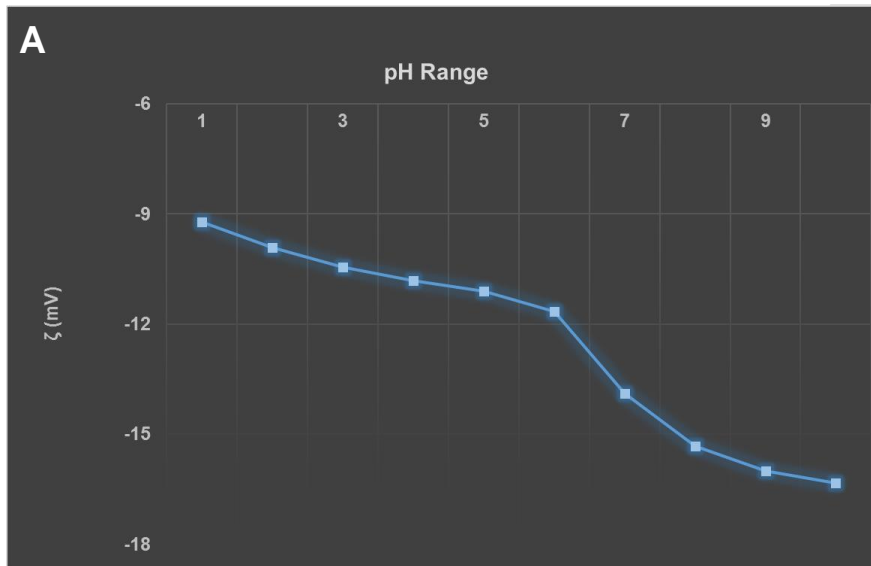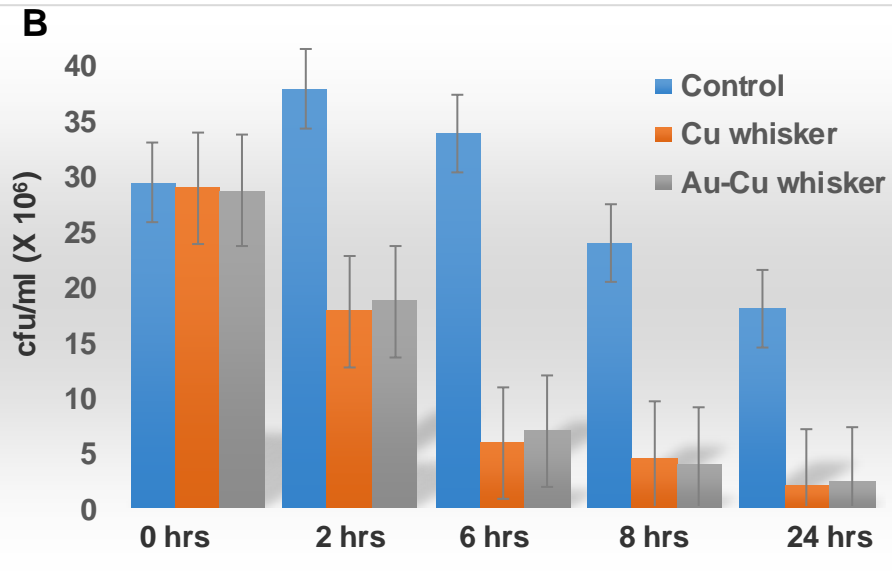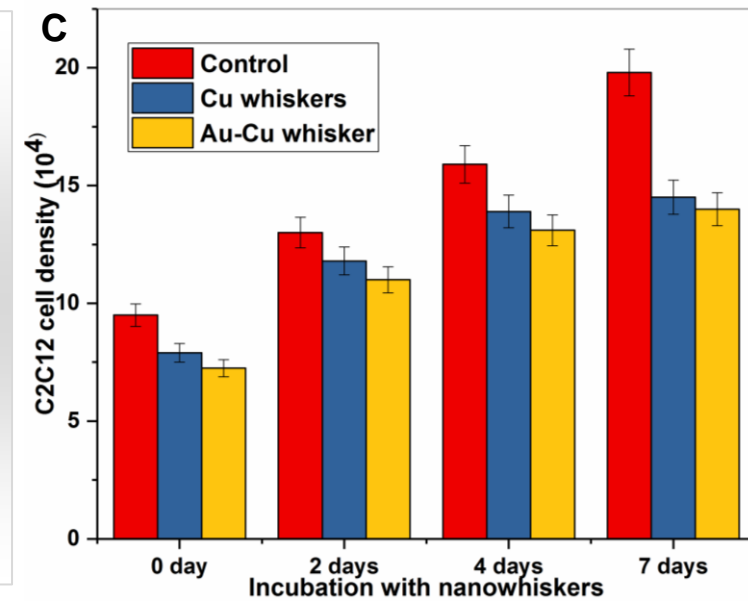

Supplement: S3 Fig — A. Zeta potential measurement of nanowhiskers. B. Graph showing antimicrobial efficacy of nanowhiskers in terms of log reduction of CFU/mL. C. Assessing cytotoxicity of nanowhiskers to mouse myoblast C2C12 cell lines. (PDF) [file pone.0175428.s003.pdf]

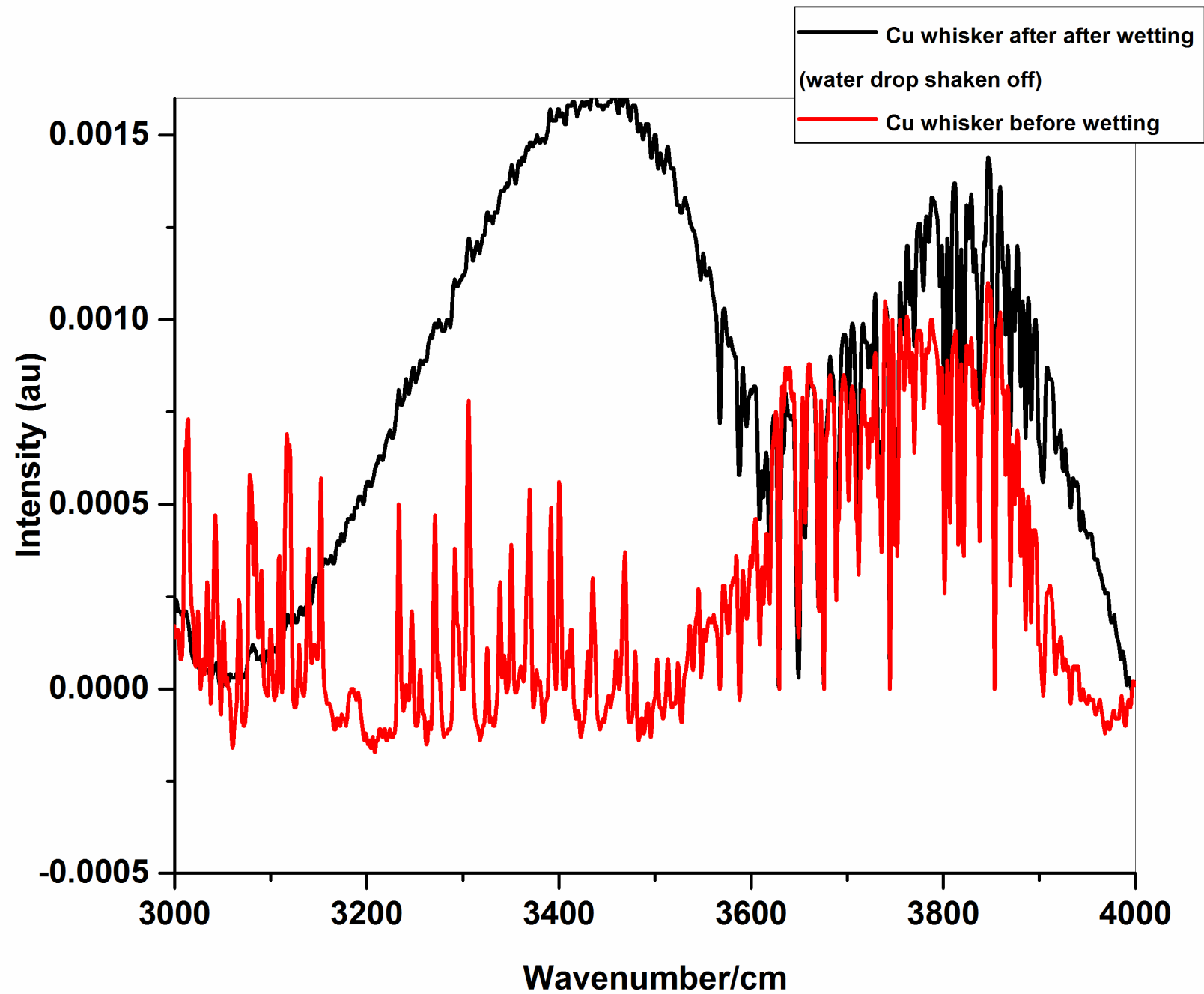

Supplement: S5 Fig — Blue line plot shows FTIR analysis before applying water drops, indicating atmospheric water vapors. Red curve represents FTIR spectra taken immediately after “pinned” water drop was forcibly shaken off the surface and spectra recorded. Black curve are recorded after 8 hours of samples were kept at room temperature. Reduced intensity exhibit some amount of residual water might had evaporated. (PDF) [file pone.0175428.s005.pdf]
