# Supplementary material for: Hydrophobic pinning with copper nanowhiskers leads to bactericidal properties
Source: PLoS One. 2017 Apr 11;12(4):e0175428. doi: 10.1371/journal.pone.0175428 (PMC5388474; doi:10.1371/journal.pone.0175428)
Supplement: S4 Fig — (PDF) [file pone.0175428.s004.pdf]

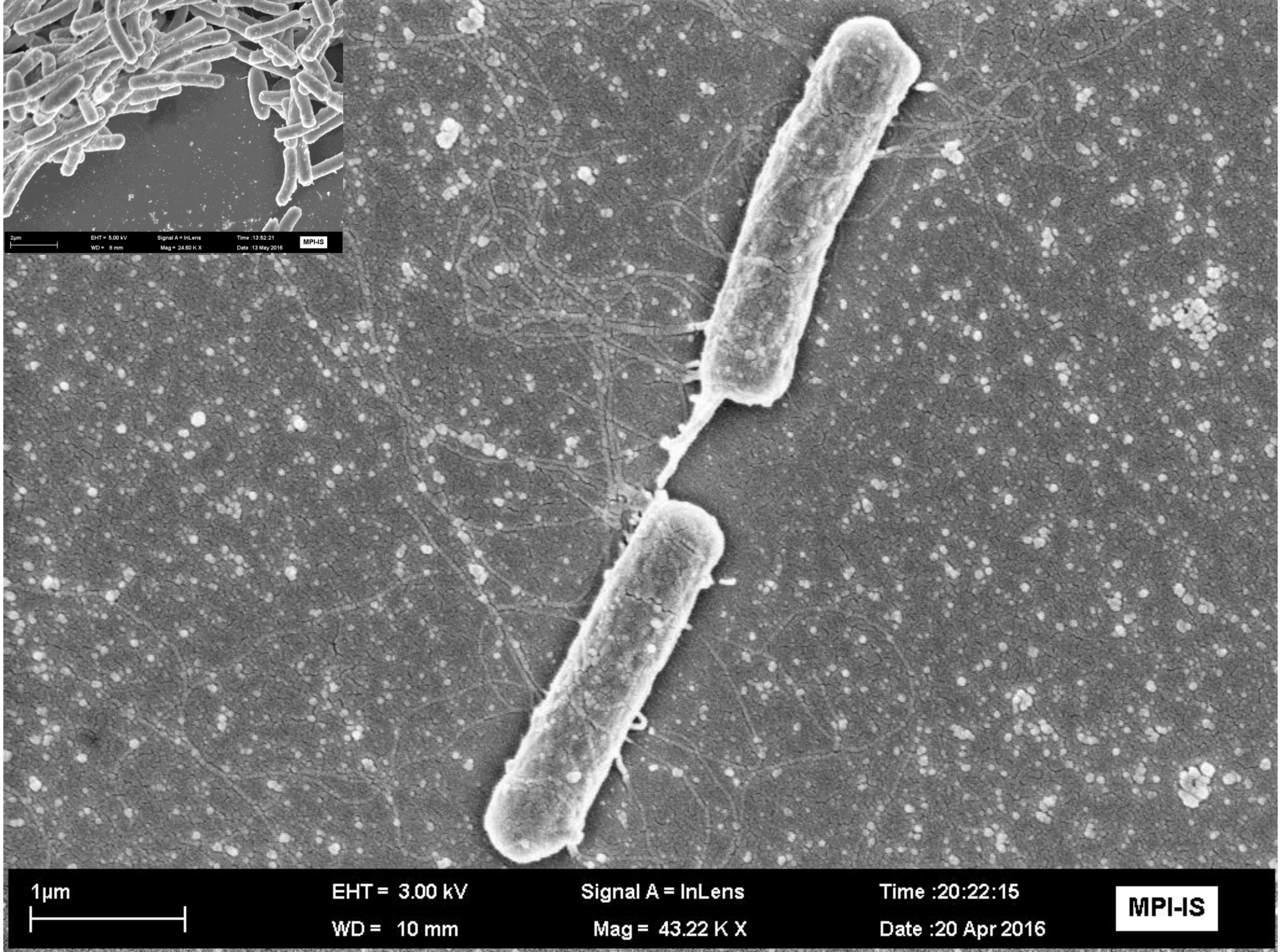
2µm
EHT = 5.00 kV
WD = 8 mm
Signal A = InLens
Mag = 24.60 K X
Time :13:52:21
Date :13 May 2016
MPI-IS
1µm
EHT = 3.00 kV
WD = 10 mm
Signal A = InLens
Mag = 43.22 K X
Time :20:22:15
Date :20 Apr 2016
MPI-IS
